# Understanding and using AlloSure donor derived cell-free DNA

**DOI:** 10.1007/s12551-020-00713-5

**Published:** 2020-07-18

**Authors:** R. K. Seeto, J. N. Fleming, S. Dholakia, B. L. Dale

**Affiliations:** 1 University of Sydney, Sydney, NSW Australia; 2 Medical University of South Carolina College of Pharmacy, Charleston, SC USA; 3 University of Oxford, Oxford, UK; 4 Vanderbilt University, Nashville, TN USA

**Keywords:** Kidney transplant, Donor derived cell free DNA, Subclinical inflammation

## Abstract

Renal transplant is a lifesaving and cost-effective intervention for patients with End Stage Renal Failure. Yet it is often regarded as replacement therapy rather than a cure given the overall failure rate over time. With a shortage of organs, this global issue has been further compounded by increased incidences of obesity, hypertension and diabetes, such that the disease burden and need for transplantation continues to increase. Considering the lifetime of immunosupression in transplant patients, there will also be significant associated co-morbidities By leveraging the advances in innovation in Next Generation Sequencing, the field of transplant can now monitor patients with an optimized surveillance schedule, and change the care paradigm in the post-transplant landscape. Notably, low grade inflammation is an independent risk for mortality across different disease states. In transplantation, sub-clinical inflammation enhances acute and chronic rejection, as well as accelerates pathologies that leads to graft loss. Cell free DNA has been shown to be increased in inflammatory processes as we all as provide an independent predictor of all-cause mortality. This review considers the utility of AlloSure, a donor derived cell free DNA molecular surveillance tool, which has shown new clinical insights on how best to manage renal transplant patients, and how to improve patient outcomes.

Following the initial technical challenge of implanting an organ, maintaining the organ against a vast array of pathologies for years to come remains a colossal challenge for all clinicians working in transplantation. Drug toxicity, opportunistic infection, primary disease recurrence, and the constant battle against organ rejection are all differentials that are considered when graft dysfunction is observed, promoting a lifetime of laborious surveillance.

Current recommendations in kidney transplantation are to follow indicators of allograft damage (serum creatinine (SCr), estimated glomerular filtration rate (eGFR), and proteinuria), markers of immune activity (donor-specific antibodies (DSA), and to consider performance of protocol (surveillance) biopsies as methods to assess graft abnormalities and identify early the need to intervene (Weir and Wali 2009). However, once SCr rises or DSA or proteinuria appears, the decline in renal function is usually inevitable. All of these are lagging indicators, which occur in response to inflammation or significant graft damage, leading to chronic graft dysfunction and loss. While surveillance biopsies may be able to identify graft abnormalities early, there is evidence that nearly 62% of borderline histological change in pathology on surveillance biopsies, which resolve without treatment (Nankivell et al. 2019). They also expose patients to significant risk of iatrogenic harm with a low yield of actionable evidence.

From the moment allografts are implanted, there is persistent sub-clinical inflammation. Post-transplant graft inflammation impairs the induction of tolerance and enhances acute and chronic rejection (Braza et al. 2016). Increased inflammation has also demonstrated an independent association with death with a functioning graft (Molnar et al. 2017)_,_ as well as increased risk of graft loss (Dahle et al. 2011; Abedini et al. 2009). As the current lagging indicators are poorly sensitive, subclinical inflammation can lead to the progression of fibrosis and chronic humoral rejection as well as the formation of DSAs (Fig. 1). The ability to identify and quantify inflammation earlier in the pathologic course can better risk stratify transplant patients in need of intervention (Torres et al. 2014).

**Fig. 1:**
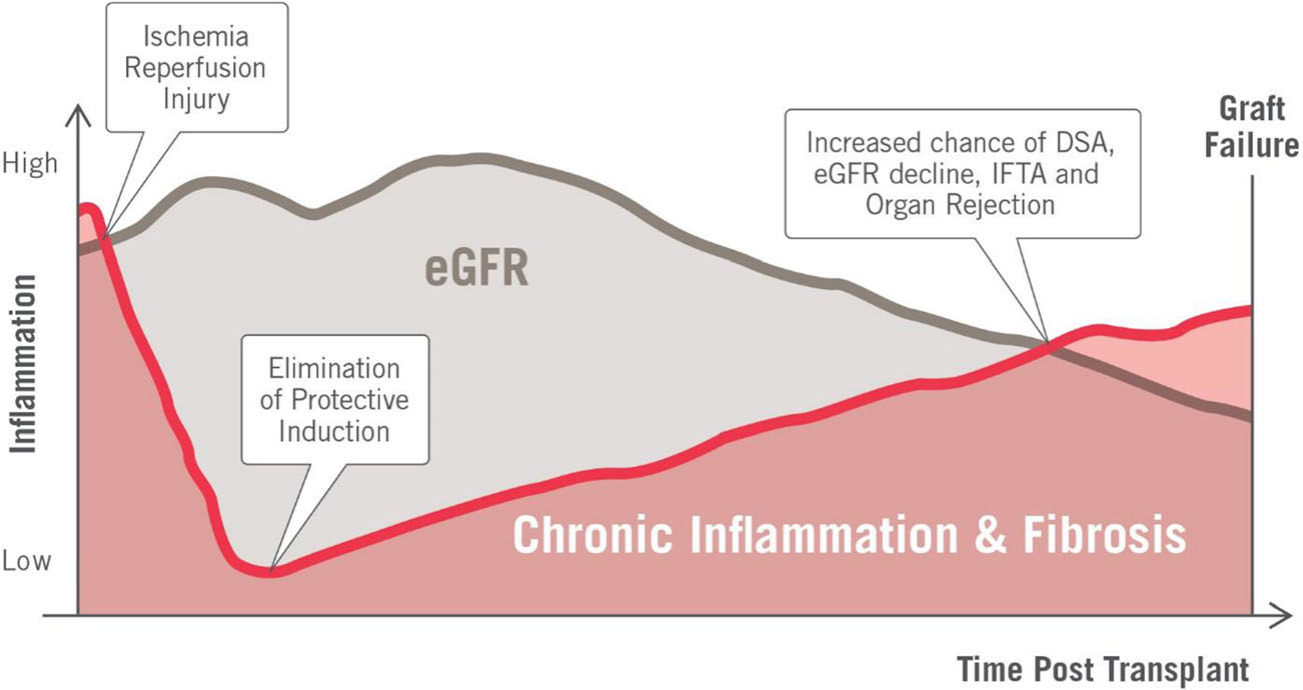
Showing the time course post-transplant of inflammation, function, and contributors of injury leading to graft failure. The progression of inflammation and injury can be quantified by AlloSure dd-cfDNA

Across all disease states, low-grade inflammation is an independent risk for mortality (Bonaccio et al. 2016). Proctor et al. have demonstrated that an inflammation-based prognostic score, combining high sensitivity C-reactive protein, albumin, and neutrophil count, is prognostic of all-cause mortality (Proctor et al. 2015). As a biomarker of injury, end-stage renal disease (ESRD) patients with increased circulating cell-free DNA (cfDNA) have also been shown to have increased inflammatory processes and increased mortality, where cfDNA is an independent predictor of all-cause mortality (Tovbin et al. 2012).

## Classification of allograft rejection

Allograft rejection is also intertwined with inflammation, resulting in specific pathologic changes, with or without graft dysfunction. This occurs due to the recipient’s immune system recognizing the non-self-antigen in the allograft and leads to progressive tissue damage. In this way, the precursors to organ injury can be detected using AlloSure dd-cfDNA.

Both innate and adaptive immune systems play a significant role in rejection, but the T lymphocytes (a component of the adaptive immune system) are the principal cells that recognize the allograft and amplify a response via the delayed type (type IV) hypersensitivity reaction. However, there are other costimulatory molecules and cytokines which can also play a major role in this reaction. Depending on the histopathology and immunological characteristics, renal transplant rejections can be classified broadly under the following categories:

1. Hyperacute rejection: This happens within minutes after transplant and has been directly linked to preformed antibody to the human leukocyte antigens, the tissue, or ABO incompatibility; this is rarely seen now due to the very sensitive crossmatch tests performed before the transplant.
2. Acute rejection: This can occur any time after transplant, usually within days to weeks after transplant. It classifies into the following:
  (A) Antibody-mediated rejection (ABMR): which usually demonstrates evidence of circulating donor-specific alloantibodies and immunological evidence of antibody-mediated injuries to the kidney, such as complement deposition, inflammation of glomeruli (glomerulitis) or peritubular capillary (peritubular capillaritis).
  (B) Acute T cell-mediated rejection (TCMR): which is characterized by varying amounts of lymphocytic infiltration of the tubules, interstitium, and, in advanced stages, the arterial intima.
3. Chronic rejection: This usually develops more than 3 months post-transplant and can either be chronic ABMR or chronic TCMR.
4. Mixed rejection: This occurs when multiple immunological pathways are queued and results in acute rejection superimposed on chronic rejection.

## The burden and cost of allograft rejection

ABMR is the leading cause of allograft dysfunction and loss after kidney transplantation. The detection of DSA was previously required as a prerequisite to diagnose ABMR; however, more recently, there is recognition that ABMR that occurs is the absence of detectable DSA. Therefore, other molecular markers of injury, including dd-cfDNA detected by AlloSure, are increasingly being used as substitutes for DSA. The Banff working group in 2017 accepted molecular assays as substitutes for DSA when diagnosing ABMR (Haas et al. 2018).

Sussell et al. have shown that, despite improvements in outcomes for kidney transplant recipients in the past decade, graft failure continues to impose substantial burden on patients (Sussell et al. 2020). In this study, authors compared outcomes from a simulation model of kidney transplant patients, in which patients who were at risk for graft failure were compared with an alternative simulation in which the risk of graft failure is assumed to be zero. Transitions through the model were estimated using Scientific Registry of Transplant Recipients (SRTR) data from 1987 to 2017. Lifetime costs, overall survival, and quality-adjusted life years (QALYs) for both scenarios were analyzed and compared to obtain the burden of graft failure.

Within this study, the average patient with graft failure will impose additional medical costs of $78,079 (95% confidence interval [CI] $41,074, $112409) and a loss of 1.66 QALYs (95% CI 1.15, 2.18). Given 17,644 kidney transplants in 2017, the total incremental lifetime medical costs associated with graft failure is $1.38B (95% CI $725M, $1.98B) and the total QALY loss is 29,289 (95% CI 20291, 38,464). Thus, efforts to reduce the incidence of graft failure or to mitigate its impact are urgently needed.

The current treatment options for ABMR, its clinical and economic burden, and approaches for reducing the risk of ABMR are of grave concern as ABMR is responsible for up to 60% of late graft failures. While ABMR is notoriously resistant to treatment with corticosteroids, additional approaches have been used over the evolution and understanding of pathogenic antibodies such as depletion, inhibition, or neutralization of DSA. These partially effective treatments do not come without a cost, which can range between reported costs of USD $49,000–$155,000 per episode (Muduma et al. 2016).

Unfortunately, leaving ABMR untreated is not an alternative option as it places patients at high risk for adverse events which may ultimately result in a return to dialysis (Reyna-Sepulveda et al. 2017). Given the cost and inefficiency of treatment, interventions targeting prevention of ABMR are critical. Preventing nonadherence to immunosuppressants is a key strategy; however, it has proved an exceedingly difficult target given the dynamic process of nonadherence and lack of accurate real-time community-wide monitoring method.

It has become evident that a more sensitive marker or predictor of allograft injury is required to identify at-risk patients early enough that meaningful clinical intervention may even be possible.

### Donor Derived Cell Free DNA

Since its discovery in 1948, cfDNA has made an impactful change in transplantation. A growing body of evidence (109 manuscripts from 55 studies) and clinical use has demonstrated that donor-derived cell-free DNA (dd-cfDNA), as an early and accurate detector of allograft injury, provides a quantitative marker of inflammation as part of screening and routine monitoring during the post-transplant period (Knight et al. 2019; Sherwood and Weimer 2018; Thongprayoon et al. 2020).

dd-cfDNA itself has also been identified as a trigger of inflammation, thereby adding insult to injury (Dholakia et al. 2020). Being able to identify early un-resolving molecular allograft injury measured via changes in dd-cfDNA allows the stratification of patients who are at risk of subsequent allograft injury, immunological activity, or declining graft function (Jordan et al. 2018; Clayton et al. 2016). Considering dd-cfDNA as a continuous and clinically significant biomarker for kidney transplant opens the potential for new management strategies, optimizing clinical decisions and the potential for improved clinical outcomes.

Clinical validity for dd-cfDNA in plasma has been shown by numerous studies showing the value of dd-cfDNA in the surveillance in kidney transplant recipients (Sigdel et al. 2018; Bloom et al. 2017).

The Circulating Donor-Derived Cell-Free DNA in blood for diagnosing Acute Rejection in Kidney Transplant Recipients (DART) study (ClinicalTrials.gov Identifier: NCT02424227) assessed 1272 blood specimens from 384 kidney recipients from 14 clinical sites at scheduled post-transplant intervals (Bloom et al. 2017). The median levels of dd-cfDNA in kidney transplant recipients with active rejection were significantly higher (1.6%) than in the comparator group (0.3%) of biopsy specimens without active rejection (*p* < 0.001) with a receiver-operating characteristic (ROC) area under the curve (AUC) of 0.74. At 1.0% dd-cfDNA, there was an 85% specificity and 59% sensitivity to discriminate active rejection from no rejection. The PPV was 61% and NPV was 84%, at 1.0% dd-cfDNA.

The ROC plot for ABMR had an AUC of 0.87. At 1.0% dd-cfDNA, there was an 83% specificity and 81% sensitivity to discriminate ABMR from no ABMR. The PPV was 44% and NPV was 96.4% at 1.0% dd-cfDNA for ABMR vs no ABMR. Median dd-cfDNA was 2.9% (ABMR), 1.2% (TCMR, types ≥ IB), 0.2% (TCMR type IA), and 0.3% (controls); *p* < 0.001, ABMR vs controls; *p* = 0.05, TCMR type ≥ IB vs controls.

**Fig. 2:**
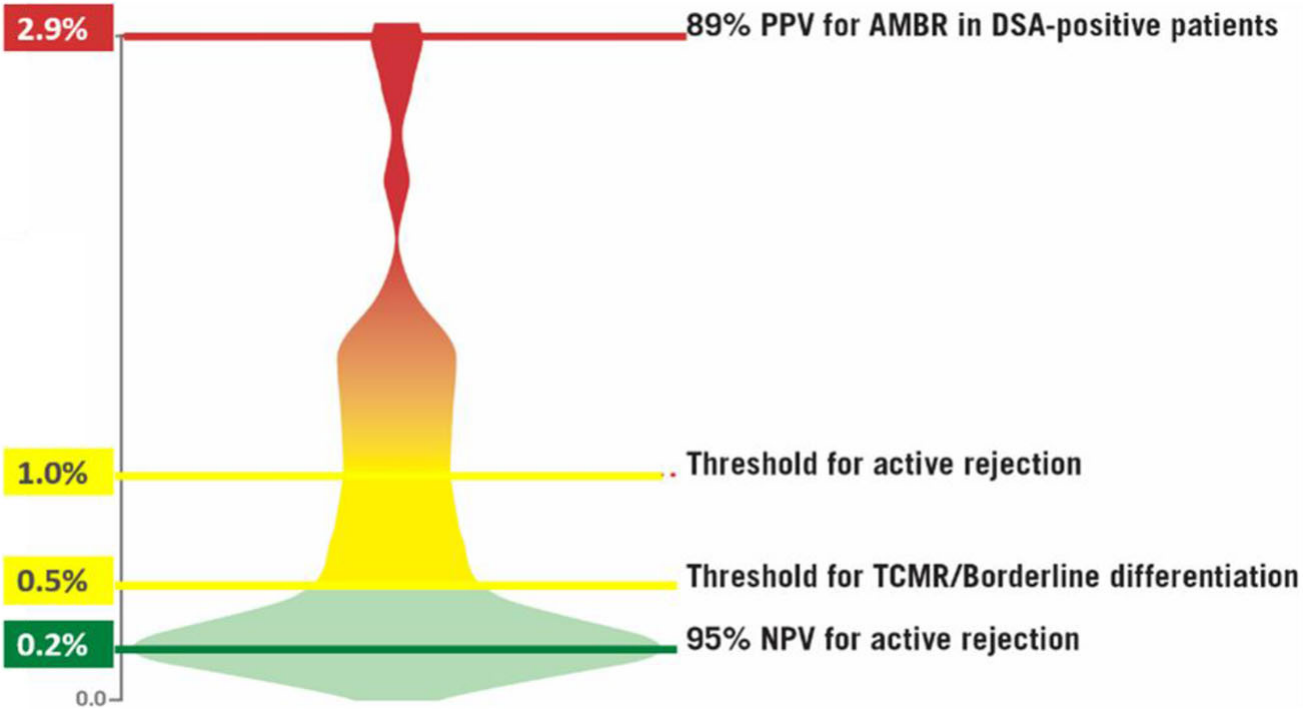
Violin plot, showing the continuum of AlloSure across the population. The negatively skewed distribution shows how the change in AlloSure in combination with the absolute number supports clinical decisions at different levels

In the DART study, most (204/242) kidney transplant biopsies were clinically indicated; yet, only 27% of these clinically indicated biopsies revealed active rejection, and so, optimizing the use of renal biopsy using AlloSure is important.

Huang et al. further showed clinical validation of AlloSure in a single-center experience (Huang et al. 2019). A total of 485 samples from 352 adult kidney transplant patients were assessed with AlloSure. Sixty-three patients had a paired allograft biopsy; 27 patients (43%) had donor-specific antibodies (DSA) and 34 patients (54%) had biopsy-proven rejection. There were 22 patients with isolated ABMR, 10 with TCMR, and 2 with mixed rejection. The study found that AlloSure was able to distinguish patients with antibody-mediated rejection (ABMR) from those with no rejection (median dd-cfDNA 1.35% vs 0.38%, respectively; *p* < 0.001). The AUC-ROC was 0.82 (95% CI: 0.71–0.93) for distinguishing ABMR from no rejection. With a dd-cfDNA cutoff value of 0.74%, AlloSure detected ABMR with 100% sensitivity, 71.8% specificity, 68.6% PPV, and 100% NPV.

From the moment the allograft is implanted, there is a continuous release of dd-cfDNA into the recipient’s circulation.

Therefore, considering AlloSure as a continuum rather than a discrete threshold is important.

Stites et al. published a violin pilot (Fig. 2) showing the gradient in the relative change in AlloSure levels at the time of biopsies showing borderline changes or TCMR grade 1A, demonstrating the continuum of dd-cfDNA as allograft damage progresses (Stites et al. 2020). The same way clinicians trend blood pressure, creatinine, or changes in drug levels, changes in AlloSure provide insight into the directionality of allograft injury. Bromberg et al. have shown that results of 0.2% are associated with immune quiescence and give clinicians a peace of mind score, with a high NPV, showing that there is no significant injury to the allograft (Bromberg et al. 2017). Stites et al. published results above 0.5% are associated with progression to clinical outcomes when measured at the time of biopsies demonstrating borderline changes or TCMR1A rejection, which is supported by Huang et al. who showed that 0.74% was associated with allograft rejection. Bloom et al. published level at 1% and higher are associated with allograft rejection (Sigdel et al. 2018; Bloom et al. 2017), which continue to increase in PPV as levels continue to rise.

Dd-cfDNA values greater than 1.2% are above the 97.5th percentile in a study of stable kidney transplant recipients and therefore are outside the normal range for this population. Additionally, an increase of <61% in a consecutive dd-cfDNA value in an individual is achange that may be attributable to normal biological variation (Bromberg et al. 2017).

#### Donor-specific antibodies

DSA have become an established biomarker predicting ABMR and has a prevalence of around 15–20% within the first year of transplantation. Everly et al. reported that 11% of the patients without detectable DSA at the time of transplantation will have detectable DSA 1 year later, and over the next 4 years, the incidence of de novo DSA will increase to 20%. After de novo DSA development, 24% of allografts will fail within 3 years (Everly et al. 2013).

Preformed DSAs in sensitized patients can trigger hyperacute rejection, accelerated acute rejection, and early acute ABMR (Terasaki and Cai 2005). Transplant patients with DSA have twice the graft failure rate as those without and those without DSA. Additionally, patients without DSA have a superior graft survival 4 years post-transplant compared to those with DSA. It is now further understood that while not all DSA carry equal immunogenicity, depletion or reduction of DSA can result in increased allograft survival (Terasaki 2012; Willicombe et al. 2012).

The pathogeneses of ABMR include not only complement-dependent cytotoxicity, but also complement-independent pathways of antibody-mediated cellular cytotoxicity and direct endothelial activation and proliferation. The novel assay for complement binding capacity has improved our ability to predict and stratify potentially pathogenic antibodies from less-harmful antibody (Chen et al. 2011). C1q fixation is a classic marker of the complement-dependent pathway and, thus, is a surrogate marker for the ability of antibody to signal through this pathway. This stratification and classification of DSA can be even further enhanced by DSA sub-typing as some classes of antibody are more efficient at fixing complement than others (Zhang 2018). C1q binding donor-specific antibodies are closely associated with acute ABMR, more severe graft injuries, and early graft failure, whereas C1q nonbinding donor-specific antibodies correlate with subclinical or chronic ABMR and late graft loss (Ponsirenas et al. 2018).

Jordan et al. have shown that dd-cfDNA identifies ABMR in DSA-positive kidney transplant recipients. The authors also demonstrated that higher dd-cfDNA levels are associated with de-novo DSA formation, elucidating how prospective dd-cfDNA monitoring, with immunosuppression augmentation in response to dd-cfDNA levels, may be beneficial in patients deemed “at-risk” in the absence of clinical symptoms or biopsy findings. DSA mean fluorescence intensity (MFI) had a positive correlation with dd-cfDNA levels (*r* = 0.30 CI − 0.083 to 0.466 *p* = 0.004), with 99% of the MFIs being greater than 500. Logistic modeling with multivariate analysis showed that dd-cfDNA is independently a predictor of de-novo DSA, when controlling for race, age, donor type, and, prior transfusion, and HLA mismatch (*p* < 0.001). Importantly, in the instance, a transplant recipient has a positive biopsy for ABMR in the presence of DSA AlloSure holds an 89% PPV, at a 2.9% threshold (Jordan et al. 2018).

Further data from Stites et al. revealed that elevated levels of dd-cfDNA predicted adverse clinical outcomes. Among patients with elevated cfDNA, de novo DSA formation was seen in 40% (17/42) vs 2.7% with low AlloSure levels (*P* < 0.0001) and patients with elevated AlloSure dd-cfDNA went on to have future or persistent rejection that occurred in 9 of 42 patients (21.4%) vs 0% (*P* = 0.003) (Stites et al. 2020).

The relationship of dd-cfDNA detecting DSA has also been published in other organ transplants such as heart and lung transplantation. Kobashigawa et al. showed that the average dd-cfDNA for the patients with DSA was 1.2%, which was significantly higher than patients without DSA (0.4%). The mean DSA level, defined as MFI, was 6984 ± 4460 (MFI range 5000 to 17,500) (Kobashigawa et al. 2019). The authors concluded that dd-cfDNA appears to be correlated to the development of DSA. Furthermore, it suggests that DSA may be injuring the donor organ and may necessitate treatment of these patients. Additionally, Agbor-Enoch and Jackson et al. showed that elevated dd-cfDNA was an early risk factor for the development and persistence of de novo donor-specific HLA antibody in lung transplantation (Agbor-Enoh et al. 2018).

## BK nephropathy and viremia

Between 10 and 30% of kidney transplant recipients (KTR) develop BK viremia, with 1–10% of KTR developing BK virus-associated nephropathy (BKVAN), accounting for 7% of all renal allograft failures (Hirsch et al. 2005). BK viremia is also associated with increased risk for de novo DSA (Patel et al. 2016). Sawinski et al. explored this phenomenon and interestingly found that while persistent BK viremia was associated with formation of de novo class II DSA, after a median of 3 years post-transplant showed no difference in allograft survival (Sawinski et al. 2015). The diagnosis of BKVAN currently requires biopsy confirmation. The primary management, immunosuppression reduction, has limited efficacy, and patients often require re-biopsy to assess disease progression or resolution. Improved methods to diagnose BKVAN and follow disease progression or resolution are needed.

In a retrospective analysis of the DART study, of the 102 biopsies performed with paired dd-cfDNA, 10 patients with BK viremia or BKVAN had 14 paired dd-cfDNA and renal biopsy results, performed between 2015 and 2018. Seven KTR had BKV PCR titers that were correlated to dd-cfDNA results and biopsy pathology findings. Analysis showed a positive correlation of dd-cfDNA and BK viral load. Correlation identified an *r* value = 0.874 (95% CI 0.35–0.98, *p* = 0.01). Additionally, those patients with BK viremia without BKVAN had a median dd-cfDNA = 0.58% (IQR 0.43–1.15), while BKVAN had a median dd-cfDNA = 3.38% (IQR 2.3–4.56). KTR with biopsies meeting Banff criteria for acute cell-mediated rejection (TCMR; >Banff 1A) had a median BK PCR load = 4.42 × 10^5^ (IQR 2.1 × 10^3^–5 × 10^5^) while KTR not meeting criteria had median PCR load = 3.71 × 10^4^ (IQR 1 × 10^5^–2.2 × 10^7^), these were not statistically different (*p* = 0.45). Yet, five of seven BKVAN patients, but only two of seven with isolated viremia, had biopsies meeting Banff criteria for TCMR, with median dd-cfDNA in non-rejection patients = 0.43% (IQR 0.29–0.91) versus 2.84% (IQR 1.49–4.29) in rejection patients, *p* = 0.001 (Brennan et al. 2019).

## eGFR decline

Clinical trials designed to investigate the effectiveness of interventions on allograft loss or death of renal transplant recipients are challenging as these tend to be events which occur long-term. Therefore, surrogate markers are necessary. The decline in eGFR is commonly used as a surrogate for hard outcomes in kidney transplantation. Clayton et al. examined 7949 transplants performed from 1995 to 2009, including 71,845 patient-years of follow-up, 1121 graft losses, and 1192 deaths. Percentage change in eGFR between years 1 and 3 after transplant was examined where *a* ≥ 30% decline in eGFR, which were associated with subsequent death (hazard ratio, 2.20; 95% confidence interval, 1.87 to 2.60) and death-censored graft failure (hazard ratio, 5.14; 95% confidence interval, 4.44 to 5.95) (Clayton et al. 2016).

Additional surrogate markers were assessed in this study including acute rejection, doubling of SCr level, and eGFR at year 1 or year 2. A 30% decline in eGFR was considered superior. The authors also concluded that 30% decline in eGFR between years 1 and 3 after kidney transplant is common and strongly associated with risks of subsequent death and death-censored graft failure, which mirrors findings in CKD (Clayton et al. 2016). Faddoul et al. reported results from clinical trials in organ transplantation (CTOT) 17 also identifying a 40% decrease in post-kidney transplant eGFR from 6 months post 2 years post-transplant as a surrogate for 5-year outcomes (Faddoul et al. 2018).

Based on these data, the DART investigators assess whether increases in dd-cfDNA could be a predictor of second year eGFR decline. Of the 384 patients, 173 patients had AlloSure dd-cfDNA and eGFR measured 1–10 times during the first-year post transplant and 1–6 times during follow-up visits during the second year. The mean eGFR results from years 1 and 2 were compared in patients with ≥ 1 elevated dd-cfDNA (AlloSure ≥ 1%) in year 1 vs. those < 1% dd-cfDNA elevation. Association between elevated dd-cfDNA (≥ 1%) and the future occurrence of a low eGFR below a target level of 15–30 mL/min/1.73 m^2^ was also tested. Seventy-three percent of patients with high first year dd-cfDNA (≥ 1%) had a significant drop in eGFR in year 2 (median eGFR change − 25%, IQR − 46% to + 2%) compared to 45% patients without elevated dd-cfDNA (median eGFR change + 2%, IQR − 18% to + 45%), *p* = 0.002. This study summarized that dd-cfDNA ≥ 1% was indeed associated with eGFR < 30 mL/min (*p* = 0.040) and was a significant risk factor for a 30% decline in eGFR in the Cox model (*p* = 0.047), with a hazard ratio of 2.31 (95% CI 1.01–5.28) (Alhamad et al. 2019).

Continuing with this trend, elevated levels of dd-cfDNA (AlloSure ≥ 0.5%) in patients with TCMR1A predicted adverse clinical outcomes. Stites et al. found among patients with elevated cfDNA, eGFR rate declined by 8.5% vs 0% in low dd-cfDNA (AlloSure < 0.05%) patients (*p* = 0.004) (Stites et al. 2020).Recent publications compared different dd-cfDNA and found that although dd-cfDNA is similar, they are not the same, and so, assessing diagnostic test characteristics and clinical evidence on the supporting platform is important. As more data is generated, cross walking published dd-cfDNA data across different platforms is likely to be ineffective as different dd-cfDNAs, although similar are not the same (Dengu 2020). With the wide adoption of dd-cfDNA and the potential for further assays entering the field, a clear understanding of the technology and evaluation of real-life patient validation data supports the importance to remain consistent to a single platform and consistent surveillance schedules.
